# Gut Microbiome in Retina Health: The Crucial Role of the Gut-Retina Axis

**DOI:** 10.3389/fmicb.2021.726792

**Published:** 2022-01-14

**Authors:** Gianluca Scuderi, Emidio Troiani, Angelo Maria Minnella

**Affiliations:** ^1^Ophthalmology Unit, NESMOS Department, St. Andrea Hospital, Sapienza University of Rome, Rome, Italy; ^2^Cardiology Unit, State Hospital, Institute for Social Security, Cailungo, San Marino; ^3^Department of Ophthalmology, Fondazione Policlinico Universitario A. Gemelli-IRCCS, Catholic University of the Sacred Heart, Rome, Italy

**Keywords:** age-related macular degeneration, gut-retina axis, leaky-gut, microbiota, microbiome, micronutrient, personalized medicine

## Abstract

The term microbiome means not only a complex ecosystem of microbial species that colonize our body but also their genome and the surrounding environment in which they live. Recent studies support the existence of a gut-retina axis involved in the pathogenesis of several chronic progressive ocular diseases, including age-related macular disorders. This review aims to underline the importance of the gut microbiome in relation to ocular health. After briefly introducing the characteristics of the gut microbiome in terms of composition and functions, the role of gut microbiome dysbiosis, in the development or progression of retinal diseases, is highlighted, focusing on the relationship between gut microbiome composition and retinal health based on the recently investigated gut-retina axis.

## Introduction

The terms microbiota and microbiome are often used interchangeably, although they have different meanings: the first refers to the prokaryotic organisms (bacteria and archaea) and eukaryotes (e.g., protozoa, fungi, algae, and multicellular parasites) that coexist in symbiosis with us, the latter, which for a long time was only referred to these microorganisms genomes, today takes a broader meaning. The term microbiome means a complex ecosystem of microbial species that colonize our body, their genome, and the surrounding environment in which they live (Marchesi and Ravel, 2015).

In other words, the microbiome refers to both microorganisms and their “theater” of activity which also includes microbial metabolites, mobile genetic elements (transposons, phages, and viruses) where they form a real ecological niche. Therefore, the microbiome is considered a micro-ecosystem integrated into a macro-ecosystem represented by the host with whom it interacts in a crucial way (Berg et al., 2020).

Understanding the role of the microbiome, particularly the one belonging to the gastrointestinal (GI) tract, represents one of the most active fields of biomedical research. Microbiome composition constitutes a fundamental aspect if we want to understand its physiology and pathological implications. It comprises a microbial community that exceeds 100 trillion microorganisms, distributed with a density of 1011–1012 per milliliter. To get an idea of the biological role of this population, just think that, while the human genome consists of approximately 23,000 genes, the gut microbiome encodes more than 3 million genes and produces thousands of metabolites. It is, therefore, evident that, since it interacts in a symbiotic way with our organism, performing immune and metabolic functions of fundamental importance, the maintenance of its qualitative and quantitative composition is essential for the maintenance of our health (Rinninella et al., 2019).

This review aims to underline the importance of the gut microbiome in relation to ocular health. After a brief introduction on the gut microbiome in terms of composition and function, the review highlights the role of gut microbiome dysbiosis in the development or progression of chronic retinal diseases, such as Age-Related Macular Degeneration (ARMD), Diabetic Retinopathy (DR), or Retinitis Pigmentosa (RP). Based on the recently investigated gut-retina axis, this excursus will focus on the relationship between gut microbiome composition and retinal health.

## The Gut Microbiome

The human GI tract is one of the largest interfaces (250–400 m^2^) between the host, antigens, and environmental factors in the human body. The human gut microbiome varies taxonomically and functionally in each of the anatomical regions of the GI tract as these have different characteristics in terms of physiology, pH, oxygen tension, digestive flow rate, availability of substrates, and host secretion (Flint et al., 2012).

From the duodenum to the rectum, an increasing quantitative microbial gradient and a decreasing qualitative microbial gradient occur, also showing a progressive reduction of aerobic bacteria in favor of obligate anaerobes (Clarke et al., 2019).

New techniques based on molecular biology technologies allowed identifying the microbial phyla present in the intestinal microbiome and quantifying them, by analyzing the DNA and RNA extracted from fecal samples. It was understood that the gut microbiome comprises a few phyla such as Firmicutes, Bacteroidetes, Actinobacteria, Proteobacteria, Fusobacteria, and Verrucomicrobia, with the two phyla, Firmicutes and Bacteroidetes, representing more than 80–90% of entire gut microbiome.

The Firmicutes phylum is made up of more than 200 different genera, including *Lactobacillus*, *Bacillus, Enterococcus*, and *Ruminococcus*. Among them, the *Clostridium* genus represents 95% of this phylum. The Bacteroidetes phylum is instead composed mainly of the genera *Bacteroides* and *Prevotella*. The Actinobacteria phylum is less represented and consists prevalently of the *Bifidobacterium* genus (Rinninella et al., 2019).

## The First 1,000 Days

Individual variations hallmark gut microbiome composition due to conditioning factors related to birth, age, environment, use of antibiotics, diet (Thursby and Juge, 2017). The microbiome is, in fact, a dynamic ecosystem that varies from individual to individual and that changes over time even within the same subject.

Several studies both on animal models and humans suggest that gut colonization from the microbiota is critical during early life because this period is important for the definition of the immune system and physiological development (Koleva et al., 2015). The gestational age at birth seems to be the main factor among those affecting the first composition of the microbiota, followed by the mode of delivery (cesarean or natural), feeding and weaning but also family lifestyle, geographical location, genetics of the infant as well as the use of antibiotics (Zhuang et al., 2019).

The diversity of the microbiome changes rapidly during childhood, especially during the first 1,000 days (3 years) of life and during puberty, and then stabilizes by assuming a composition that will also be maintained in adults and which, in healthy individuals, remains mostly stable (Kundu et al., 2017). However, it remains vulnerable and can change throughout life due to several factors, including age, diet, lifestyle, drug use, etc (Ottman et al., 2012).

The microbiome of each subject is unique and regulates several physiologic functions of the host, including metabolism and development, and maintenance of immune homeostasis. Although the research in this field is still preliminary, it is well documented that a gut microbial imbalance may be responsible for dysfunctions affecting the host, contributing to the pathogenesis and/or progression of a wide spectrum of pathologies affecting various organs and systems (Kho and Lal, 2018).

## Microbiome and Immune Function: A Mutual Regulation

The close physiological link between host and microbiome has several metabolic outcomes, and a specific effect on the host immune systems. Instead, a large body of evidence indicates that several microbial metabolites profoundly regulate the immune system via host receptors and other target molecules. The relation between the microbiome and the host is at least partly mediated by metabolites synthesized by microbes which, acting as signal molecules, regulate the neuro-immune-inflammatory axis of the host. This link physiologically connects the intestine to other organs and systems (Kim, 2018). In this scenario, the immune system plays a key role. Immune cells express metabolite-specific receptors and other molecular targets that, together, provide an extensive array of signaling able to respond in different ways based on nutritional changes, health, and immunological status (Kim, 2018). Microbial metabolites strengthen the barrier tissues and train the immune system to prevent any infections by pathogens. In other words, the microbiome (through its metabolites) is essential for the correct development of the innate and adaptative immune response (Round and Mazmanian, 2009). The hematopoietic and non-hematopoietic cells of the innate immune system are strategically located at the host–microbiome interface. These cells are capable of translating signals produced by the microbiome into host response. It may explain why the altered communication between the innate immune system and the gut microbiota might contribute to complex pathologies. The emerging idea is that the gut microbiome “educates” the immune system to be both reactive to pathogens and tolerant (immunotolerance) (Thaiss et al., 2016). Our ability to discriminate between commensals and pathogenic organisms is tightly dependent on mutualism with the microbial population. The symbiotic microbes protect us from pathogens through different strategies. These strategies include a preferential consumption of nutrients necessary for their survival, small metabolites production [some Short Chain Fatty Acids (SCFAs)] limiting its growth, the negative modulation of virulent genes expression, bactericidal or bacteriostatic substances production such as bacteriocins, and conferring an immune-mediated resistance to the host against pathogens (Mezouar et al., 2018). The tight interaction between the microbiome and immune function is also due to several microbial metabolites, which have their major receptors in the immune system. Many of them act as signals for different cells and contribute to the hormone secretion as well as to the regulation of metabolism and the host’s immune system (Debnath et al., 2021). The gut microbiota can metabolize a broad spectrum of nutrients such as proteins, lipids, carbohydrates, bile acids, plant-derived molecules, and contaminants. These products are metabolized into polyamines, indoles, SCFAs, adenosine triphosphate, phytochemical metabolites, and bile acid metabolites. In particular, SCFAs act as histone deacetylase inhibitors to regulate gene expression and activate G-protein-coupled receptors; other metabolites collectively activate nuclear receptors, and other receptors such as pregnane X receptor (PXR), Vitamin D Receptor (VDR), Liver X Receptors (LXRs), and farnesoid X receptor (FXR), Takeda G protein-coupled receptor 5 (TGR5) expressed by various cells in the innate and adaptive immune systems (Kim, 2018).

## The Link Between Dysbiosis, Leaky Gut, and Inflammation

Pathological conditions causing intestinal dysbiosis and altering the production of microbial metabolites are responsible for dysregulation of the immune system and metabolism that favors the onset of diseases with immune and chronic inflammatory components. Dysbiosis is any change to the composition of resident commensal communities leading to a reduction in the number of symbionts and/or an increase in the number of pathobionts. As the commensal microbiome regulates the maturation of the mucosal immune system, while the pathogenic microbiome causes immunity dysfunction, the imbalance between them causes a non-specific inflammation that can predispose susceptible subjects to inflammatory pathologies (Shi et al., 2017).

The gut microbiome regulates essential functions for the maintenance of general human health. It influences the immune system and homeostasis both locally (within the gut) and systemically. Therefore, in this context, a key role is played by the increased intestinal permeability (leaky gut) and the related microbial translocation. That contributes to the chronic systemic inflammation process (Rizzetto et al., 2018). The gastrointestinal epithelium represents an extensive interface with the external environment. Single epithelial cells, called enterocytes, tightly connected to each other, cover the internal surface of our intestinal mucosa. These cells provide a barrier that selectively regulates the exchanges between the inner and the outer environment of luminal toxins, antigens as well as the absorption of nutrients and water, by using both transcellular and paracellular transport mechanisms (Powell, 1981). The mucosal permeability is mainly linked to the efficiency of the tight junction that regulates the paracellular trafficking of antigens, conferring immune tolerance or immune activation in response to non-self antigens (Ahmad et al., 2017).

Compromised tight junction function is associated with several clinical intestinal and systemic conditions (Odenwald and Turner, 2013; Abraham et al., 2019). However, despite several signs of progress in understanding the composition and function of the intracellular tight junctions, the mechanisms that are regulated are not fully clarified. However, there is an accordance concerning the role played by the zonulin in the gut permeability, as it is the only physiologic intestinal permeability modulator described until now (Fasano et al., 2000; Wang et al., 2000).

Gut dysbiosis may induce the release of zonulin that provokes the crossing of the luminal content through the epithelial barrier. This is responsible for the release of pro-inflammatory cytokines that, themselves, enhance the intestinal permeability causing a vicious loop leading to a massive influx of dietary and microbial antigens triggering the activation of T cells. Depending on the host genetic predisposition, activated T cells may remain in the gastrointestinal tract, causing inflammatory diseases of the gut (Crohn’s Disease, Irritable Bowel Syndrome, Irritable Bowel Disease, and Environmental Enteric Dysfunction) or migrate to several different organs causing systemic chronic inflammatory diseases (Fasano, 2020). In other words, increased intestinal permeability due to the barrier dysfunction may cause microbial translocation, which can induce a low-grade inflammation contributing to the pathogenesis of numerous chronic diseases with inflammatory components (Fukui, 2016; Tilg et al., 2020).

Moreover, gut dysbiosis could be related to the formation of the inflammatory form of lipopolysaccharide (LPS), a glycolipid found on the outer membrane of Gram-negative bacteria formed by a lipid A (endotoxin), a non-repeating core oligosaccharide and a distal polysaccharide composed of O-antigen (Salguero et al., 2019). The release of Gram-negative bacteria LPS in the gut and its passage to the blood causes LPS-associated toxicity, i.e., endotoxemia (Guo et al., 2012). LPS could move into the circulatory system through direct diffusion due to increased intestinal permeability or via absorption by enterocytes (Boroni Moreira et al., 2012).

Lipid A then initiates a signaling cascade resulting in activation of various proinflammatory pathways (predominantly NF-κB) and increases oxidative stress upon binding to Toll-Like Receptor 4 (TLR4) (Asehnoune et al., 2004; Boutagy et al., 2016), resulting in systemic and retinal inflammation (Han et al., 2020). Inflammatory LPS has also been identified as probable culprit of cardiovascular atherogenesis (Feminò et al., 2021).

## Aging: Common Denominator Between Low-Grade Inflammation and Immunosenescence

Low-grade inflammation characterizes the aging process resulting from environmental, genetic, and epigenetic events throughout life. Indeed, typical aging features are age-related diseases that share an inflammatory pathogenetic origin. The low-grade inflammation linked to the aging process has been termed “inflammaging” and is considered a significant risk factor for morbidity and mortality in elderly people (Franceschi et al., 2000). This pro-inflammatory state is characterized by an increased expression of cytokines such as IL-6 and TNF-α and the activation of transcription factors with NF-κB. This condition seems to predispose the body to the onset of numerous age-related diseases such as Parkinson’s and Alzheimer’s diseases, but also amyotrophic lateral sclerosis, multiple sclerosis, atherosclerosis, heart disease, age-related macular degeneration, diabetes, osteoporosis, cancer, and many others (Mangiola et al., 2018). On the other hand, inflammation is a rapid response of the cells to factors that challenge the cells and tissues homeostasis and is a crucial survival mechanism. However, chronic inflammation is a detrimental process contributing to several chronic age-related diseases (Franceschi and Campisi, 2014; Scuderi et al., 2019).

Aging is defined as a process that is genetically determined and modulated by the environment. The gastrointestinal tract is involved in this process as it is related to degeneration of the enteric nervous system, alteration of intestinal motility and of the epithelial-mucosal barrier, with a relative reduction of the defense function favoring the development of gastrointestinal pathologies. In this context, immunosenescence, defined as a deterioration of the immune system, plays a key role in the aging process (Soenen et al., 2016). Moreover, aging compromises the efficiency of the immune system, leading to its gradual deterioration: immunosenescence. Aging and immunosenescence are processes closely related to the inflammatory state of the organism that is considered the major risk factor for most of the aforementioned age-associated pathologies (Ostan et al., 2008). Indeed, aging is characterized by an overgrowing disease-associated pathobionts (Stavropoulou et al., 2021).

## Immunosenescence and Inflammaging: Role in the Pathogenesis of Age-Related Retinal Diseases

Immunosenescence and “inflammaging” seem to contribute also to the development of age-related eye-diseases.

Among all the ocular tissues, the retina is considered a privileged tissue from the immune standpoint. It is protected by three layers (the inner blood-retina barrier; the outer blood-retinal barrier; and the blood-aqueous barrier), as well as by “resistance” and “tolerance” strategies that safeguard it from any insults coming from the internal and external environment. Moreover, it is also protected by its own defense systems such as microglia and the complement system to maintain retinal homeostasis (Gregerson, 1998; Streilein et al., 2002; Forrester and Xu, 2012).

The retina is highly vulnerable due to its poor renewal and repair capacity, so even minor damage can have devastating consequences. Aging negatively impacts the retina, which is at increased risk of developing various degenerative diseases such as diabetic retinopathy, glaucomatous retinopathy, and age-related macular degeneration. In fact, the three layers protecting the retina undergo significant changes during aging. Physical barriers become to be weakened due to age-related dysfunction of endothelial and glial cells. Immune regulatory signals are also altered. All the changes make the retina more susceptible to oxidative stress, hyperglycemia, and increased intraocular pressure (Chen et al., 2019).

The mechanisms responsible for the development of age-related macular degeneration are still not fully understood. However, it seems that immunosenescence (altered immune functions during aging), involves the retina compartment and plays an important role in retinal disease. Current data suggest that age-related macular degeneration is a systemic immunologic disease with local expression due to the decline of the ocular downregulatory immune environment. A sign of an altered immune response is represented by the deposits that gradually accumulate in the retinal pigment epithelium first and by the drusen later, constituting an antigenic stimulus that favors a marked activation of the immune system, especially in predisposed subjects (Nussenblatt and Ferris, 2007).

During aging, all defense systems become less efficient, and the morphofunctional and immune changes related to age are concomitant to chronic low-level inflammation (Franceschi and Campisi, 2014). The “inflammaging” process also contributes to age-related retinal diseases. Among them, age-related macular degeneration is strongly characterized by inflammatory components. Subretinal drusen resemble the extracellular deposits that characterize other degenerative diseases such as Alzheimer’s disease, amyloidosis, atherosclerosis (Mullins et al., 2000; Kaarniranta et al., 2011). They have been shown to contain many potentially harmful substances, including lipids, lipoproteins, lipofuscin, beta-amyloid, oxidation products, and inflammation-related factors such as complement components, immunoglobulins, molecules of the HLA system, and acute-phase proteins such as fibrinogen, vitronectin, and others (Anderson et al., 2002; Crabb et al., 2002; Ebrahimi and Handa, 2011). There is no solid evidence that chronic low-level inflammation and complement activation also play a decisive role in drusen formation (Johnson et al., 2001; Anderson et al., 2002). Inflammation is so much part of the disease that various inflammatory molecules, both systemic and local, have also been proposed as biomarkers to follow the evolution of the disease clinically and the response to therapy. Still, at present no specific and reliable marker has been found, and there is no univocal agreement on that (Kauppinen et al., 2016).

## Aging and Microbiome in the Gut Microbiome-Brain Axis

Aging strongly affects the composition of the microbiome because it contributes altering the intestinal epithelial barrier, whose function is fundamental to maintaining homeostasis. This barrier layer includes various elements such as the epithelium, the mucous layer, antimicrobial peptides, and IgA secretory immunoglobulins. These components are closely connected with the resident microbial community and suffer deterioration too, related to aging (Branca et al., 2019). With age, when all body functions also decline due to a gradual loss of stem cells and organ inefficiency increases due to the lower ability to repair cell damage, the composition of the microbiome also changes, reducing its diversity and richness (Reza et al., 2019). During aging we assist to a progressive modification of gut microbiota due to host factors as genetics, intestinal and immunological senescence, and due to environmental factors such as nutrition, biogeography, exposure to drugs, etc (García-Peña et al., 2017). There is no chronological threshold or age at which the composition of the microbiota suddenly alters, and changes occur gradually with time (O’Toole and Jeffery, 2015). These age-related microbial variations increase proteolytic bacteria and decrease saccharolytic bacteria (Bischoff, 2016). A reduced microbial diversity, a reduction in the species producing substances that favor the integrity of the barrier function, and an increase in potential pathogens correlate with the onset of numerous age-related diseases, including, for example, neurodegenerative ones.

The age-related microbiome alteration may affect general health Alteration in gut microbiota composition may, particularly, alter the brain aging since it is an integral part of the gut-brain axis. The interactions between the central nervous system, the enteric nervous system, and the gastrointestinal tract have been extensively studied (Flanagan et al., 2020). A series of preclinical studies have suggested a significant role in gut microbiota in these gut-brain interactions (Mayer et al., 2015). The gut microbiota-brain axis refers to a bidirectional communication network between gut and brain strongly mediated by the immune system. Its composition includes gut microbiota and their metabolic products, enteric nervous system, sympathetic and parasympathetic branches, neural-immune system, neuroendocrine system and central nervous system. Moreover, there might have five possible routes of communicating between gut microbiota and brain, including the gut-brain’s neural network, neuroendocrine-hypothalamic-pituitary-adrenal axis, gut immune system, some neurotransmitters and neural regulators synthesized by gut bacteria, and barriers including intestinal mucosal barrier and blood–brain barrier (Wang and Wang, 2016).

## Gut-Eye Axis

The health of the microbiome is essential in maintaining the human body in a homeostatic state. Aberrations in the gut microbiota and its metabolites induce systemic inflammation via stimulation of both the innate and adaptive response of the immune system. This inflammation may proceed to destroy tissues throughout the body, leading to the onset of many diseases, including the ocular ones, as shown in both human and animal studies (Floyd and Grant, 2020).

The eye is prone to develop inflammatory diseases even without an infectious component, appears to be influenced by dysbiosis of the gut microbiome. There is a, relevant association between inflammatory intestinal diseases and ocular diseases. Indeed, ten percent of subjects suffering from inflammatory bowel disease manifest ocular diseases (episcleritis, uveitis, conjunctivitis, etc.) (Vavricka et al., 2015). Several studies have shown the existence of a gut-eye axis, where gut bacteria can affect immunity at distant sites, including the eye (Kugadas et al., 2017). Moreover, other studies have highlighted that both the intestinal microbiome and its metabolites, especially the SCFA, can regulate the key functions of immune cells by modifying directly or indirectly the epigenome of different cell types (Woo and Alenghat, 2017; Wen et al., 2018). Sight-threatening immune response damaging the eye is a typical characteristic of intraocular inflammatory diseases. Uveitis, age-related macular degeneration, Sjogren’s syndrome associated with dry-eye, diabetic retinopathy, glaucoma, and infectious keratitis have been linked to gut microbiome abnormalities although the data supporting these associations are still in the early stages (Fehér et al., 2018; Cavuoto et al., 2019).

## Gut-Eye Axis and Retinal Diseases

Several retinal diseases such as ARMD, DR, and RP may be influenced by the gut microbiome suggesting a direct gut-retina axis.

ARMD is a multifactorial disease resulting from a complex combination of genetic and environmental factors. As microbiome integrity plays a crucial role in the host regulation and promotion of immunity, as discussed earlier, intestinal microbiota could represent a candidate for interplay between the genetic risk and environmental factors contributing to ARMD (Lin, 2019).

Data support that the higher translocation of gut metabolites and products permitted by the increased intestinal permeability related to an altered gut microbiota may modulate retina-specific immune cells (Andriessen et al., 2016). Interestingly chronic inflammation in response to LPS accelerates neurodegeneration in dystrophic P23H rats resulting in worsening morphological and physiological disturbances in dystrophic retinas (Noailles et al., 2018).

Zinkernagel et al. (2017) evaluated the sequenced gut metagenomes of patients with ARMD and controls in order to verify whether alteration in both composition and function of the intestinal microbiome was associated with ARMD. They observed an association between neovascular ARMD and microbiome composition at the taxonomical levels of bacterial genera and species. They found the genera *Anaerotruncus* and *Oscillibacter* as well as *Ruminococcus torques* and *Eubacterium ventriosum* were higher in patients with ARMD, whereas *Bacteroides eggerthii* were higher in controls. Authors speculate this diversity may be ARMD linked because the first genera (*Anaerotruncus* and *Oscillibacter* as well as *Ruminococcus* torques and *Eubacterium ventriosum*), increased in these patients, are associated with glutamate degradation and increased arginine biosynthesis pathways (Zinkernagel et al., 2017). Glutamate is a well know excitatory neurotransmitter in the retina, therefore its reduction may result in deficient neurotransmission in the retina (Bui et al., 2009). Furthermore, it is known that patients with increased arginine levels are frequently affected by progressive chorioretinal atrophy, which suggests a role for arginine in the development of retinal degeneration (Simell and Takki, 1973). Moreover, patients analyzed were also deficient of bacteria responsible for fatty acid elongation pathway. In this regard, it is known that long chain polyunsaturated fatty acids may have critical impact on the retinal physiology and may contribute to ARMD (Liu et al., 2010). For these reasons authors suggest that alterations in the intestinal microbiome were linked to ARMD (Zinkernagel et al., 2017). In contrast the abundance of *Bacteroides eggerthii* in controls was, probably, protective against the disease due to its ability to produce SCFAs. These metabolites might regulate intraocular inflammation probably by altering migration of lymphocytes from the intestine to the eye (Chen et al., 2021).

Dietary factors could represent a link between alteration of gut microbiota and ARMD. As reported by Rowan et al. (2017) the consumption of a “high-glycemia diet” has been associated with some typical clinical signs of the disease such as retinal pigment epithelium hypopigmentation and atrophy, accumulation of lipofuscin as well as degeneration of photoreceptors. In contrast, the consumption of a “lower-glycemia diet” did not. Instead, the switch from the first to the second type of diet could reverse the age-related macular degeneration features. A “lower-glycemia diet” limits the accumulation of advanced glycation end products, polyunsaturated lipids, and related peroxidation end-products. Metabolomic analysis revealed that gut microbiota is influenced by the diet and can be protective against the disease producing some metabolites like serotonin. Particularly, the order of *Clostridiales* was associated with “high-glycemia diet” and with related clinical features of the disease, whereas the order of *Bacteroidales* was associated with “lower-glycemia diet” and was protective against the disease (Rowan et al., 2017). These data reveal another nexus between gut microbiota and its metabolites (influenced by the diet), and the retina health, supporting the hypothesis of a gut-retinal axis.

Other than ARMD, also DR can be considered another ocular disease linked to intestinal dysbiosis. DR is one of the long-term microvascular complications of diabetes and, thus, attributable to metabolic, immune, and inflammatory alterations that characterize diabetes. Dysbiosis leads to an imbalance in the production of gastrointestinal metabolites, causing inflammatory status, alteration in glucose homeostasis, and insulin resistance. SCFAs and other substances such as trimethylamine N-oxide (TMAO), lipopolysaccharide, aromatic amino acids, and their metabolites contribute to the development of type 2 diabetes through different metabolic and immunologic pathways (Tanase et al., 2020). On the other hand, as reported by Fernandes at al., hyperglycemia seems to be responsible for some gut barrier alterations, leading to the loss of immune system homeostasis, associated with the systemic chronic inflammatory process, even involving retinal vessels. Authors hypothesize all these changes contribute to retinal inflammation, breakdown of the blood-retinal barrier, apoptosis, and neovascularization leading to diabetic retinopathy progression (Fernandes et al., 2019). Oubaha et al. (2016) report several data suggesting that inflammatory processes associated with age-related gut dysbiosis may contribute to premature senescence of retinal cells, which, through releasing pro-inflammatory cytokines and angiogenic factors, lead to neovascularization and disruption of vascular repair.

Another retinal disease influenced by the gut microbiome is the RP. Kutsyr et al. (2021) have shown, in an experimental mouse model, that changes in gut microbiome composition are linked to RP. They observed some retinal degenerative abnormalities, such as deterioration of both retinal responsiveness to light stimuli and visual acuity, in animals with retinitis pigmentosa and with changes in gut microbiome. Specifically, the *Bacteroides caecimuris*, typically less represented in the healthy gut microbiome, was significantly higher in diseased mice in comparison to control mice. On the contrary, affected mice were missing the most common genetic variants (*Rikenella spp., Muribaculaceae spp., Prevotellaceae UCG-001 spp*., and *Bacilli spp*.) typical of a healthy gut microbiome (Kutsyr et al., 2021). The link between gut microbiome changes and ocular diseases has been explained by several mechanisms (Nayyar et al., 2020). Gut dysbiosis can favor the increased intestinal permeability allowing microbes and their metabolites to induce ocular cell inflammation (Zinkernagel et al., 2017). A microbial imbalance may also be responsible for the breakdown of the blood-retinal barrier and the increased oxidative stress in the central nervous system (Li et al., 2020). All these hypotheses may also explain the neuroinflammation, oxidative stress and cell death registered in the mice model with retinitis pigmentosa. Furthermore, it can be hypothesized that different stages of retinal degeneration might be related to different changes in the gut microbiome (Kutsyr et al., 2021).

Moreover, the gut microbiota is a critical player in the metabolism and absorption of several macro- and micronutrients. It plays an important role in modulating the biochemical profile of the human diet. Bacteria and their metabolic pathways are notoriously involved in carbohydrates and protein metabolism. Gut microbiota synthesizes some vitamins such as vitamin K and B group vitamins needed for bacterial metabolism. Still, they are also important for the metabolic and physiological human pathways. Furthermore, its seems that gut microbiota may affect the metabolism of polyphenols to influence their bioactivity (Rowland et al., 2018). Finally, human and animal studies have underlined that gut microbiome composition may be influenced by several dietary nutrients such as omega-3 polyunsaturated fatty acids, which along with other nutrients (Costantini et al., 2017) are involved in ARMD (Rinninella et al., 2018; Battaglia Parodi et al., 2020).

Taken together, all the evidence supports the hypothesis of a “gut-retina axis” in the pathogenesis of retinal diseases and ARMD.

The health of the eye is linked to the health of the intestine. Therefore, it can be supposed that a better understanding of the relationship between the eye and the gut microbiome could facilitate the development of new targeted therapies for the treatment of eye disorders (Lerner et al., 2020).

## Microbiome Modulation: A New Therapeutic Target for Ocular Diseases

The intestinal microbiome plays a central role in maintaining the health of our body, and its qualitative and quantitative balance is essential to prevent the onset and/or worsening of several pathological conditions, especially with an inflammatory component. Therefore, the scientific community has begun to identify the microbiome as a critical new therapeutic target for different pathologies, including the ophthalmic ones. This close relationship between intestinal microbiota and host led to the development of an ever-growing interest in using a probiotic-based biotherapy that aims to heal the intestinal environment by balancing microbial populations (Butel, 2014; Westfall et al., 2017).

The probiotic term, according to the definition of the World Health Organization, identifies all microorganisms able to bring benefits to the health of the host. According to the most recent definition, probiotics are “live microorganisms which, when administered in an adequate amount, can confer a health benefit on the host” (Hill et al., 2014; Tsai et al., 2019). The mechanism of action of probiotics is related to their ability to compete with pathogenic microorganisms for adhesion sites, antagonize these pathogens or modulate the host’s immune response.

The use of innovative nutraceutical products, based on probiotics, capable of modulating gut microbial colonization is believed to prevent and combat several pathologies (Belizário et al., 2018). In this regard, it is important to emphasize that choosing the right probiotic in relation to the disease is not easy for many reasons. For instance, the probiotic function varies significantly within the same species; different bacterial strains of the same genus and species can have completely different effects on the host. The specific properties and characteristics of a particular bacterial strain should be well understood and the impact on the health of the host. In other words, although the use of probiotics is considered beneficial, it is necessary to choose the proper probiotic, in terms of cell numbers and formulation as clinical data are still conflicting (Sniffen et al., 2018; Suez et al., 2019).

Among the probiotic strains most used, lactic acid bacteria are widely consumed to enhance the intestinal barrier and immune function. Among these, *Lactobacillus paracasei* KW3110 has been particularly studied for its eye benefit, in experimental models and in clinical studies (Morita et al., 2018a,b, c).

This strain was evaluated for its protective effects on the blue light-induced human retinal pigment epithelium cell damage caused by visual display terminal (VDT) loads. The study showed that the ingestion of this specific strain suppressed cell death (Morita et al., 2018a).

The positive results led the researchers to further investigate the molecular mechanism responsible for the protective effect on the retina produced by the *Lactobacillus paracasei* KW3110. Therefore, they conducted experimental evaluations to observe the effect of long-term intake (6 months) of *Lactobacillus paracasei* KW3110 on age-related inflammation and altered gut microbiota in physiologically aged mice. The results showed effects on gut microbiota by modulating the age-related changes of bacterial composition, including bifidobacteriaceae strains, and the anti-inflammatory effect highlighted by the significant reduction of cytokines and chemokines in the mice treated compared to controls (IL-17, KV, and IL-13). Furthermore, *Lactobacillus paracasei* intake suppressed retinal inflammation by reducing cytokine-producing macrophages and the loss of age-related retinal cells (Morita et al., 2018b). Likewise, other experiments demonstrated the ability of the *Lactobacillus paracasei* to suppress inflammation on photoreceptor cells in a murine model of light-induced retinopathy. Although additional clinical research is needed to translate research into clinical practice, authors conclude that these data may suggest that this specific strain of *Lactobacillus paracasei* may have a preventive effect against degenerative retinal diseases (Morita et al., 2018c).

A lot of work still needs to be done to identify specific probiotic strains related to particular diseases. The microbiota modulation through probiotics or next-generation beneficial microbes constitutes a future perspective for developing either nutritional or pharmaceutical agents to maintain health.

Alongside probiotics biotherapy, there is also the use of prebiotic, a class of substances that, in 2016, have been defined by a panel of experts in microbiology, nutrition and clinical research, as “a substrate that is selectively utilized by host microorganisms conferring a health benefit” (Gibson et al., 2017). Fructooligosaccharides (FOS), inulin, and galactooligosaccharides (GOS) are considered the traditional prebiotics with the major evidence of beneficial effects on human health due to their fermentation. However, other substances may be considered with the same or greater effect than such prebiotics (Carlson et al., 2018). The beneficial effects on the host have been extensively studied, as they are capable of increasing the presence of Lactobacilli and Bifidobacteria by producing beneficial metabolites, promoting the absorption of calcium, reducing protein fermentation, pathogen bacteria, counteracting intestinal permeability, and enhancing the immune system (Carlson et al., 2018). A newly emerging area of research in functional foods also regards the use of postbiotics, a mixture of metabolic substances released by the microorganisms which, directly or indirectly, may promote health benefits on the host (Nataraj et al., 2020). These postbiotics seem to have pleiotropic effects, including immunomodulatory, anti-inflammatory, and antioxidant properties. Although some of these effects have been evaluated in clinical studies, data are still not conclusive. Therefore, their employment will require further evaluations to confirm or not their clinical potential (Żółkiewicz et al., 2020).

Individual microbiome composition can be influenced by several lifestyle factors such as dietary habits, socioeconomic status, education, medical care, and environmental factors. It is well known that drug use, lack of exercise, smoking, and an unhealthy diet may compromise the balance of the microbiome ecosystem (Martinez et al., 2021).

Among all these factors diet is, probably, the most critical as it may influence both the richness and diversity of microbiota. For example, red wine- and tea-derived polyphenols and vitamin D can modulate potentially beneficial bacteria. Recent research demonstrates that dietary fibers, such as inulin, galacto-oligosaccharides, arabinoxylans, and oligofructose, promote several beneficial bacteria and suppress potentially harmful species. Concerning macronutrients, the quantity and type of fat modulate both beneficial and potentially harmful bacteria, as well as the Firmicutes/Bacteroides ratio in the gut. Moreover, the type, amount of dietary proteins, and cooking methods have differential effects on the gut microbiota. Further clinical investigation about the role of micro- and macro-nutrients on the microbiome is needed (Yang et al., 2020).

## Future Direction and Concluding Remarks

In this review, we have described the importance of the microbiome in maintaining general health, as dysbiosis is strongly related to autoimmune, inflammatory, and metabolic diseases. We have described how commensal microbes affect all aspects of immune development and homeostasis in health and disease.

Increasing evidence demonstrates that the intestinal microbiome can influence the intestinal area and other tissues distant from the intestine, including the eye. There is a broad agreement on the existence of a gut-eye axis. Accordingly, the eye can be strongly influenced by the intestinal microbiota. Indeed, microbiota abnormalities are often associated with several eye diseases, all attributable to an alteration of the immune system and with an inflammatory component such as ARMD.

The significant role of the microbiota in modulating the physiological and pathological processes of our organism is now a consolidated concept. This opens new scenarios for promising therapeutic targets and treatment of many extra-intestinal pathologies, including ocular comorbidities.

It is becoming clear that the manipulation of the intestinal microbiota and the consequent maintenance of the physiological composition of the ocular one both seem to represent valid alternatives for the prevention and/or treatment of eye diseases. Although the clinical efficacy of probiotic therapy warrants further investigations, it represents a promising field of research with predictable exciting results.

## Author Contributions

GS, ET, and AM conceptualized and wrote the manuscript. All authors contributed to the article and approved the submitted version.

## Conflict of Interest

The authors declare that the research was conducted in the absence of any commercial or financial relationships that could be construed as a potential conflict of interest.

## Publisher’s Note

All claims expressed in this article are solely those of the authors and do not necessarily represent those of their affiliated organizations, or those of the publisher, the editors and the reviewers. Any product that may be evaluated in this article, or claim that may be made by its manufacturer, is not guaranteed or endorsed by the publisher.
